# Reporting antimicrobial susceptibilities and resistance phenotypes in *Staphylococcus* spp.: a nationwide proficiency study

**DOI:** 10.1093/jac/dkab017

**Published:** 2021-02-08

**Authors:** Felipe Fernández-Cuenca, Inmaculada López-Hernández, Emilia Cercenado, Carmen Conejo, Nuria Tormo, Concha Gimeno, Alvaro Pascual

**Affiliations:** Unidad Clínica de Enfermedades Infecciosas, Microbiología Clínica y Medicina Preventiva, Hospital Universitario Virgen Macarena, Sevilla, Spain; Instituto de Biomedicina de Sevilla (IBIs), Hospital Universitario Virgen Macarena/CSIC/Universidad de Sevilla, Sevilla, Spain; Spanish Network for the Research in Infectious Diseases (REIPI RD16/0016), Instituto de Salud Carlos III, Madrid, Spain; Unidad Clínica de Enfermedades Infecciosas, Microbiología Clínica y Medicina Preventiva, Hospital Universitario Virgen Macarena, Sevilla, Spain; Instituto de Biomedicina de Sevilla (IBIs), Hospital Universitario Virgen Macarena/CSIC/Universidad de Sevilla, Sevilla, Spain; Spanish Network for the Research in Infectious Diseases (REIPI RD16/0016), Instituto de Salud Carlos III, Madrid, Spain; Servicio de Microbiología y Enfermedades Infecciosas, Hospital General Universitario Gregorio Marañón, Madrid, Spain; Departamento de Medicina, Facultad de Medicina, Universidad Complutense, Madrid, Spain; CIBERES, Centro de Investigación Biomédica en Red de Enfermedades Respiratorias, CB06/06/0058, Madrid, Spain; Instituto de Biomedicina de Sevilla (IBIs), Hospital Universitario Virgen Macarena/CSIC/Universidad de Sevilla, Sevilla, Spain; Spanish Network for the Research in Infectious Diseases (REIPI RD16/0016), Instituto de Salud Carlos III, Madrid, Spain; Departamento de Microbiología, Universidad de Sevilla, Sevilla, Spain; Servicio de Microbiología, Hospital General de Valencia, Valencia, Spain; Servicio de Microbiología, Hospital General de Valencia, Valencia, Spain; Unidad Clínica de Enfermedades Infecciosas, Microbiología Clínica y Medicina Preventiva, Hospital Universitario Virgen Macarena, Sevilla, Spain; Instituto de Biomedicina de Sevilla (IBIs), Hospital Universitario Virgen Macarena/CSIC/Universidad de Sevilla, Sevilla, Spain; Spanish Network for the Research in Infectious Diseases (REIPI RD16/0016), Instituto de Salud Carlos III, Madrid, Spain; Departamento de Microbiología, Universidad de Sevilla, Sevilla, Spain

## Abstract

**Objectives:**

To evaluate the proficiency of microbiology laboratories in Spain in antimicrobial susceptibility testing (AST) of *Staphylococcus* spp.

**Materials and methods:**

Eight *Staphylococcus* spp. with different resistance mechanisms were selected: six *Staphylococcus aureus* (CC-01/*mecA*, CC-02/*mecC*, CC-03/BORSA, CC-04/MLSBi, CC-06/*blaZ* and CC-07/linezolid resistant, *cfr*); one *Staphylococcus epidermidis* (CC-05/linezolid resistant, 23S rRNA mutation); and one *Staphylococcus capitis* (CC-08/daptomycin non-susceptible)*.* Fifty-one laboratories were asked to report: (i) AST system used; (ii) antimicrobial MICs; (iii) breakpoints used (CLSI or EUCAST); and (iv) clinical category. Minor, major and very major errors (mEs, MEs and VMEs, respectively) were determined.

**Results:**

The greatest MIC discrepancies found were: (i) by AST method: 19.4% (gradient diffusion); (ii) by antimicrobial agent: daptomycin (21.3%) and oxacillin (20.6%); and (iii) by isolate: CC-07/*cfr* (48.0%). The greatest error rates were: (i) by AST method: gradient diffusion (4.3% and 5.1% VMEs, using EUCAST and CLSI, respectively); (ii) by breakpoint: 3.8% EUCAST and 2.3% CLSI; (iii) by error type: mEs (0.8% EUCAST and 1.0% CLSI), MEs (1.8% EUCAST and 0.7% CLSI) and VMEs (1.2% EUCAST and 0.6% CLSI); (iii) by antimicrobial agent: VMEs (4.7% linezolid and 4.3% oxacillin using EUCAST); MEs (14.3% fosfomycin, 9.1% tobramycin and 5.7% gentamicin using EUCAST); and mEs (22.6% amikacin using EUCAST).

**Conclusions:**

Clinical microbiology laboratories should improve their ability to determine the susceptibility of *Staphylococcus* spp. to some antimicrobial agents to avoid reporting false-susceptible or false-resistant results. The greatest discrepancies and errors were associated with gradient diffusion, EUCAST breakpoints and some antimicrobials (mEs for aminoglycosides; MEs for fosfomycin, aminoglycosides and oxacillin; and VMEs for linezolid and oxacillin).

## Introduction

*Staphylococcus aureus* is one of the most common causes of community- and hospital-acquired infections and is associated with high morbidity and mortality. The annual epidemiological report for 2017 conducted by the ECDC showed that *S. aureus* was the second most common cause of ICU-acquired pneumonia and that CoNS were the leading cause of ICU-acquired bloodstream infections (23.6%).^1^

Both *S. aureus* and CoNS can also become resistant to many of the antimicrobial agents used in clinical practice. One of the most important antimicrobial resistance mechanisms described in *S. aureus* is the acquisition of methicillin resistance mediated by the *mecA* gene and, less frequently, by the *mecC* gene.^2–4^ Methicillin resistance among *S. aureus* can also be due to hyperproduction of β-lactamases and, in some cases, to point mutations in PBP genes, leading to the so-called borderline oxacillin-resistant *S. aureus* (BORSA).^2^^,^^3^^,^^5^^,^^6^ The rates of MRSA in Europe vary considerably, ranging from 0% (Iceland) to 43% (Romania).^7^ Other resistance mechanisms reported less frequently than oxacillin resistance, but still having a great clinical and epidemiological impact, include: acquisition of 23S rRNA methylases encoded by a variety of *erm* genes, with either constitutive or inducible expression, that lead to cross-resistance to macrolides, lincosamides and streptogramin B agents (MLSBs); linezolid resistance due to either mutations in 23S rRNA or acquisition of the chloramphenicol-florfenicol resistance gene (*cfr*) coding for a methyltransferase able to methylate ribosomes and decrease linezolid binding; and alterations of the cell surface charge conferring daptomycin resistance.^8–12^

Antimicrobial susceptibility testing (AST) of staphylococci presents certain challenges related to several factors, such as species identification, acquired resistance mechanisms, types of antimicrobial discs (e.g. antimicrobial activity may vary significantly depending on the manufacturer and disc content) or the methodology used (e.g. broth microdilution versus disc diffusion).^13–15^ Another factor that can influence the susceptibility result is the interpretation criteria used (CLSI or EUCAST).^16–17^

It is possible to infer the mechanisms of resistance to antimicrobials in staphylococci as part of the interpretative reading of the antibiogram for epidemiological purposes.^18–19^ Such inference may be particularly difficult when (i) several resistance mechanisms are present (e.g. fluoroquinolone and aminoglycoside resistance), (ii) the mechanism of resistance is unknown or not well understood (e.g. resistance to daptomycin), (iii) the expression of resistance is inducible (e.g. clindamycin resistance induced by macrolides) or (iv) resistance is heterogeneous (e.g. oxacillin or vancomycin).

These challenges led us to perform the present study with the aim of identifying the most frequent problems found in AST of *Staphylococcus* spp. in Spanish clinical microbiology laboratories.

## Materials and methods

### Bacterial isolates, species identification and AST

Eight *Staphylococcus* spp. isolates with different mechanisms of antimicrobial resistance were selected for this study (Table 1). Isolates were coded from CC-01 to CC-08: CC-01 (*S. aureus*/*mecA*), CC-02 (*S. aureus*/*mecC*), CC-03 (*S. aureus*/BORSA), CC-04 (*S. aureus*/inducible MLSB resistance), CC-05 (*Staphylococcus epidermidis*/linezolid resistant, 23S rRNA mutation), CC-06 (*S. aureus*/penicillin resistant, *blaZ* positive), CC-07 (*S. aureus*/linezolid resistant, *cfr* positive) and CC-08 (*Staphylococcus capitis*/daptomycin non-susceptible). The resistance mechanism of each isolate was not revealed to the participating laboratories. All the isolates tested, except for CC-02, were collected from patients admitted to the University Hospital Gregorio Marañón (Madrid, Spain). Isolate CC-02/*S. aureus mecC* was a clinical isolate obtained from a patient at the University Hospital Lucus Augusti (Lugo, Spain).

**Table 1.** MICs (mg/L) of 21 antimicrobials against eight *Staphylococcus* spp. isolates obtained by the reference laboratories

Antimicrobial	CC-01 (*S. aureus*) *mecA*	CC-02 (*S. aureus*) *mecC*	CC-03 (*S. aureus*) BORSA	CC-04 (*S. aureus*) MLSBi	CC-05 (*S. epidermidis*) 23S rRNA mutation	CC-06 (*S. aureus*) *blaZ*	CC-07 (*S. aureus*) *cfr*	CC-08 (*S. capitis*) Daptomycin non-susceptible
Oxacillin	**>32**	**4**	2	**16**	**>32**	0.25	**>32**	0.25
Cefoxitin^a^	**64**	**16**	4	**32**	**128**	0.5	**512**	**1**
Benzylpenicillin	**128**	**2**	**2**	**>32**	**16**	**2**	**32**	**0.5**
Vancomycin	1	1	0.5	0.5	1	0.5	1	4
Teicoplanin	0.25	0.25	0.5	≤0.125	2	0.25	1	4
Linezolid	2	2	2	1	**256**	2	**64**	2
Daptomycin	0.12	0.12	0.12	0.25	0.12	0.12	0.12	**4**
Erythromycin	**64**	0.25	**64**	**>128**	**8**	0.125	**>512**	0.25
Clindamycin	≤0.06	0.12	≤0.06	0.06	**2**	≤0.06	**>512**	0.125
Ciprofloxacin	**2**	0.5	**16**	0.25	**32**	0.125	**>32**	0.25
Levofloxacin	0.5	0.25	**8**	0.25	**8**	0.12	**>32**	0.125
Co-trimoxazole	0.06	0.06	0.125	0.06	**8**	0.06	0.125	0.06
Rifampicin	≤0.003	≤0.015	≤0.003	≤0.015	**128**	≤0.015	0.015	0.015
Gentamicin	≤0.05	0.25	0.5	0.5	**>64**	0.125	**>128**	0.03
Tobramycin	0.5	0.25	1	0.5	**>64**	0.25	**64**	0.25
Amikacin	16	2	16	2	**128**	2	8	1
Tetracycline	**32**	0.5	0.25	0.25	1	0.25	0.125	0.125
Chloramphenicol	4	8	4	4	**128**	8	**>128**	0.25
Fusidic acid	0.06	0.06	1	0.06	**8**	0.06	0.06	0.06
Quinupristin/dalfopristin	≤0.25	0.12	0.25	0.25	0.125	0.12	**4**	0.25
Fosfomycin	≤8	≤8	≤8	≤8	≤8	≤8	**>64**	≤8

MICs shown in bold correspond to the R clinical category using EUCAST breakpoints version 8.1 whereas MICs in normal type correspond to the S category.

a No EUCAST breakpoints are defined for cefoxitin and those defined by CLSI have been used.

Bacterial identification, antimicrobial susceptibility and confirmation of the mechanisms of resistance were verified independently by two clinical microbiology reference laboratories: Hospital Universitario Virgen Macarena (Seville, Spain) and Hospital General Universitario Gregorio Marañón (Madrid, Spain). Identification was performed by conventional microbiological tests and MALDI-TOF (Bruker Daltonics, Madrid, Spain). The antimicrobials tested were oxacillin, cefoxitin, penicillin G, vancomycin, teicoplanin, linezolid, daptomycin, erythromycin, clindamycin, ciprofloxacin, levofloxacin, co-trimoxazole, rifampicin, gentamicin, tobramycin, amikacin, tetracycline, chloramphenicol, fusidic acid, quinupristin/dalfopristin and fosfomycin. All the antimicrobials except fosfomycin were tested in duplicate at each reference centre by disc diffusion and broth microdilution, according to EUCAST and CLSI guidelines.^20^^,^^21^ MICs of fosfomycin were determined by agar dilution. The 2018 EUCAST and CLSI breakpoints were used for the interpretation of clinical categories.^20^^,^^21^

### Study design

Isolates were sent in Amies transport medium to 52 participating hospitals in October 2018. One hospital did not send the results required and was not included in the study, so the actual number of participating hospitals was 51. Six results obtained using the Wider I system, which is currently off the market, and four results obtained by Sensititre were excluded from the study as results from these two AST systems were not representative (<0.1% of all the MICs reported).

The instructions specified that isolates should be treated as blood culture isolates. Participating laboratories were requested to fill in an electronic form for each isolate, which included: (i) the laboratory system or method used for AST; (ii) the antimicrobial susceptibility results [inhibition zone diameters or MIC values, and clinical category: susceptible (S), intermediate (I), if using CLSI guidelines, and resistant (R)]; (iii) the breakpoints used (CLSI or EUCAST); and (iv) any inferred mechanism(s) responsible for the observed phenotype of resistance.

### Data analysis

The analysis of results consisted of: (i) a descriptive analysis of AST methods, breakpoints applied and clinical category assigned; (ii) an analysis of discrepancies in MICs; (iii) an analysis of categorical error rates [minor errors (mEs), major errors (MEs) and very major errors (VMEs)]; and (iv) the ability of participating laboratories to accurately infer possible underlying resistance mechanisms.^22^

We considered that there was a discrepancy in the MIC value of any antimicrobial tested when the MIC provided by the participating laboratory was not within a single 2-fold dilution (±1 doubling dilution) of the reference result.

## Results

### Type of AST system

Fifty out of the 51 participating laboratories performed AST using an MIC-based system (5075 MIC determinations reported), whereas only 1 centre used the disc diffusion method. The percentages of MIC determinations obtained using the different quantitative AST systems were as follows: 64.8% MicroScan WalkAway (Beckman Coulter Inc., Brea, CA, USA); 28.4% VITEK 2 (bioMérieux, Marcy-l’Étoile, France); 3.3% in-house broth microdilution; 2.1% gradient strips [1.9% Etest^®^ (bioMérieux) and 0.2% Liofilchem^®^ (Waltham, MA, USA)]; and 1.4% Phoenix (BD Biosciences, Sparks, MD, USA).

With respect to the AST method used (Table 2), the discrepancies in MIC results ranged from 19.4% (gradient strips) to 6.8% (Phoenix). The least reliable of the AST systems tested were gradient strips and in-house broth microdilution. Using gradient strips, the discrepancies were associated with high VMEs (4.3% using EUCAST and 5.1% using CLSI), mainly with linezolid and daptomycin. Using in-house broth microdilution, the more relevant MIC discrepancies were associated with high MEs (4.8% using EUCAST), these MEs being associated with oxacillin. For VITEK 2, MicroScan and Phoenix, in contrast to gradient strips and in-house broth microdilution, the discrepancies observed were associated with low VMEs (≤1.4%), MEs (≤1.7%) and mEs (≤1.4%). MicroScan was the least reliable automated AST system, with the highest VMEs (e.g. oxacillin and linezolid), MEs (e.g. tobramycin, gentamicin and oxacillin) and mEs (e.g. amikacin). Using VITEK 2, the errors were as follows: VMEs (e.g. erythromycin), MEs (e.g. oxacillin and erythromycin) and mEs (e.g. oxacillin and erythromycin). Using Phoenix, there was only one VME with linezolid and one ME with oxacillin.

**Table 2.** Distribution of discrepancies in MIC results and categorical error rates obtained using different AST systems for *Staphylococcus* spp.

AST system	No. of MICs reported (no. of centres)^a^	Discrepant MICs (%)^b^	No. of MICs interpreted using		Categorical errors (%)^c^							
					Overall		mEs		MEs		VMEs	
			EUCAST	CLSI	EUCAST	CLSI	EUCAST	CLSI	EUCAST	CLSI	EUCAST	CLSI
Gradient diffusion	108 (23)	**19.4**	69	39	4.3	**5.1**	0.0	0.0	0.0	0.0	**4.3**	**5.1**
VITEK 2	1439 (17)	**11.8**	892	547	2.0	2.6	0.6	1.1	0.7	1.3	0.7	0.2
MicroScan	3289 (33)	9.8	2477	812	3.8	2.0	0.9	0.7	1.7	0.4	1.2	0.9
Broth microdilution	166 (3)	8.4	21	145	**4.8**	2.1	0.0	1.4	**4.8**	0.7	0.0	0.0
Phoenix	73 (1)	6.8	73	0	1.4	0.0	0.0	0.0	0.0	0.0	1.4	0.0

a Number of MIC determinations reported for each AST system. The number of laboratories reporting MICs for each AST system is indicated in parentheses. The 21 antimicrobials were not tested by all five AST methods. Thirteen out of the 33 laboratories using MicroScan and 10 out of the 17 laboratories using VITEK 2 reported the MICs of some antimicrobials, particularly vancomycin, linezolid or daptomycin, using gradient strips.

b Percentages of discrepancies in the MICs. MICs >1 dilution from the reference values were considered discrepant. MIC discrepancies >10% are highlighted in bold.

c The highest percentages of overall errors, as well as mEs >10%, MEs >3% and VMEs >1.5%, are highlighted in bold.

### Discrepancies and categorical errors by type of antimicrobial agent and breakpoint applied

MIC discrepancies ranged from 21.3% (daptomycin) to 0.0% (chloramphenicol) and were unacceptably high (>10%) for daptomycin, oxacillin, fosfomycin, clindamycin and penicillin G (Table 3). The discrepancies in the MICs of daptomycin, oxacillin, fosfomycin, clindamycin, tobramycin, quinupristin/dalfopristin, gentamicin, amikacin, linezolid and tetracycline produced an unacceptably high percentage of categorical errors (Tables 3 and 4). In contrast, the discrepancies in the MICs of the remaining antimicrobials produced very low categorical errors (erythromycin, fusidic acid, levofloxacin, rifampicin and cefoxitin) or did not produce any errors (vancomycin, teicoplanin, co-trimoxazole, ciprofloxacin and chloramphenicol) (Tables 3 and 4).

**Table 3.** Distribution of discrepancies in MIC values of 21 antimicrobials and categorical error rates for *Staphylococcus* spp.

Antimicrobial	No. of MICs reported (no. of centres)^a^	Discrepant MICs (%)^b^	No. of MICs interpreted using		Categorical errors (%)^c^							
					Overall		mEs		MEs		VMEs	
			EUCAST	CLSI	EUCAST	CLSI	EUCAST	CLSI	EUCAST	CLSI	EUCAST	CLSI
Daptomycin	329 (49)	**21.3**	223	106	1.7	0.0	0.0	0.0	0.4	0.0	1.3	0.0
Oxacillin	374 (50)	**20.6**	258	116	6.6	3.4	0.4	0.0	1.9	3.4	**4.3**	0.0
Fosfomycin	27 (6)	**18.6**	14	13	**14.3**	NA	0.0	NA	**14.3**	NA	0.0	NA
Clindamycin	355 (51)	**13.5**	243	112	3.2	4.5	0.4	0.9	1.2	0.0	**1.6**	**3.6**
Penicillin G	293 (44)	**13.3**	192	101	1.0	0.0	0.5	0.0	0.0	0.0	0.5	0.0
Vancomycin	364 (49)	9.1	248	116	0.0	0.0	0.0	0.0	0.0	0.0	0.0	0.0
Tobramycin	246 (35)	8.9	187	59	**9.6**	NA	0.0	NA	**9.1**	NA	0.5	NA
Erythromycin	357 (51)	8.4	248	109	2.4	7.3	1.2	7.3	0.4	0.0	0.8	0.0
Quinupristin/ dalfopristin	37 (7)	8.1	19	18	5.3	**16.7**	5.3	0.0	0.0	**11.1**	0.0	**5.6**
Gentamicin	341 (46)	7.6	244	97	5.7	2.1	0.0	2.1	**5.7**	0.0	0.0	0.0
Amikacin	99 (20)	7.1	84	15	**25.0**	NA	**22.6**	NA	2.4	NA	0.0	NA
Linezolid	370 (51)	7.0	256	114	5.1	4.4	0.0	0.0	0.4	0.9	**4.7**	**3.5**
Fusidic acid	155 (26)	5.8	104	51	1.0	NA	0.0	NA	0.0	NA	1.0	NA
Co-trimoxazole	305 (43)	5.6	203	102	0.0	0.0	0.0	0.0	0.0	0.0	0.0	0.0
Levofloxacin	292 (44)	4.1	219	73	1.4	4.1	0.0	1.4	0.9	2.7	0.5	0.0
Rifampicin	232 (36)	4.3	140	92	1.4	3.3	0.0	2.2	1.4	1.1	0.0	0.0
Tetracycline	144 (25)	4.9	111	33	4.5	3.0	1.8	0.0	0.9	0.0	**1.8**	**3.0**
Cefoxitin	172 (31)	2.9	137	35	1.5	0.0	0.0	0.0	0.0	0.0	1.5	0.0
Teicoplanin	322 (46)	1.9	224	98	0.0	0.0	0.0	0.0	0.0	0.0	0.0	0.0
Ciprofloxacin	213 (29)	0.9	143	70	0.0	0.0	0.0	0.0	0.0	0.0	0.0	0.0
Chloramphenicol	48 (9)	0.0	35	13	0.0	0.0	0.0	0.0	0.0	0.0	0.0	0.0
Overall (%)	5075	10.5	3532	1543	3.8	2.3	0.8	1.0	1.8	0.7	1.2	0.6

NA, not available.

a Number of MIC determinations reported for each AST system. The number of laboratories reporting MICs for each AST system is indicated in parentheses. The 21 antimicrobials were not tested by all five AST methods. Thirteen out of the 33 laboratories using MicroScan and 10 out of the 17 laboratories using VITEK 2 reported the MICs of some antimicrobials, particularly vancomycin, linezolid or daptomycin, using gradient strips.

b Percentages of discrepancies in the MICs. MICs >1 dilution from the reference values were considered discrepant. MIC discrepancies >10% are highlighted in bold.

c The highest percentages of overall errors, as well as mEs >10%, MEs >3% and VMEs >1.5%, are highlighted in bold.

**Table 4.** Contribution of discrepant MICs in the production of categorical errors using EUCAST breakpoints for *Staphylococcus* spp.

Antimicrobial^a^	No. of discrepant MICs^b^	No. of discrepant MICs in the range of the clinical category of			No. of discrepant MICs producing categorical errors^c^			
		S	R	I	Overall errors	VMEs	MEs	mEs
Daptomycin	70	67	3	0	**4/4**	**3/3**	**1/1**	NA
Oxacillin	77	67	10	0	14/16	10/11	4/5	NA
Fosfomycin	5	0	5	0	1/2	NA	1/2	NA
Clindamycin	48	0	27	21	3/8	0/4	**3/3**	0/1
Penicillin G	39	3	36	0	1/3	1/2	NA	0/1
Tobramycin	22	1	21	0	13/18	0/1	13/17	NA
Erythromycin	30	23	1	5	**6/6**	**2/2**	**1/1**	**3/3**
Gentamicin	26	1	25	0	**14/14**	NA	**14/14**	NA
Amikacin	7	0	5	2	5/21	NA	**2/2**	3/19
Linezolid	26	16	10	0	**13/13**	**12/12**	**1/1**	NA
Fusidic acid	9	1	8	0	**1/1**	**1/1**	NA	NA
Levofloxacin	12	6	6	0	**3/3**	**1/1**	**2/2**	NA
Rifampicin	10	2	8	0	1/2	NA	1/2	NA
Tetracycline	7	4	2	1	4/5	**2/2**	**1/1**	1/2
Cefoxitin	5	5	0	0	**2/2**	**2/2**	NA	NA

NA, not applicable.

a Antimicrobials for which discrepant MICs did not produce any errors are not shown.

b MICs >1 dilution from the reference values were considered discrepant.

c The numerator of the fraction indicates the number of errors produced only by discrepant MICs. The denominator indicates the number of errors produced by discrepant MICs (MICs >1 dilution from the reference values) and non-discrepant MICs (MICs within ±1 dilution from the reference values). Errors produced only by discrepant MICs are in bold.

As shown in Table 4, for daptomycin, oxacillin, erythromycin and cefoxitin, more than 75% of the discrepant MICs that caused categorical errors were in the susceptible category (MIC underestimation) using EUCAST breakpoints. The discrepant MICs of daptomycin, erythromycin and cefoxitin were responsible for all the VMEs and MEs, whereas discrepant MICs of oxacillin were responsible for 10/11 VMEs and 4/5 MEs (Table 4). In contrast, for fosfomycin, tobramycin, gentamicin, amikacin and rifampicin, more than 70% of the discrepant MICs that caused categorical errors were in the resistant category (MIC overestimation) using EUCAST breakpoints (Table 4). The discrepant MICs of gentamicin and amikacin were responsible for all the MEs, whereas the discrepant MICs of fosfomycin and rifampicin were responsible for 1/2 of the MEs. The discrepant MICs of tobramycin were responsible for 0/1 of the VMEs and 13/17 of the MEs. With respect to the discrepant MICs producing mEs, amikacin was the antimicrobial associated with the fewest mEs (3/19).

Although more than 70% of the discrepant MICs of penicillin G and fusidic acid that caused errors were in the resistant category, they did not produce MEs.

The discrepant MICs of clindamycin, linezolid, levofloxacin and tetracycline that caused errors were in the resistant category (56.3% for clindamycin, 50.0% for levofloxacin, 38.5% for linezolid and 28.6% for tetracycline) and the formerly intermediate category, known at present as ‘susceptible, increased exposure’ (14.3% for tetracycline and 43.8% for clindamycin) (Table 4). These discrepant MICs produced all of the VMEs for linezolid, tetracycline and levofloxacin, and all of the MEs for clindamycin, levofloxacin, linezolid and tetracycline.

Non-discrepant MICs (MICs within ±1 dilution from the reference values) that were associated with categorical errors occurred with amikacin (16/21), clindamycin (5/8), fosfomycin (1/2), penicillin G (2/3), tobramycin (5/18), tetracycline (1/5) and oxacillin (2/16) (Table 4). These non-discrepant MICs were responsible for VMEs (4/4 for clindamycin, 1/1 for tobramycin and 1/11 for oxacillin), MEs (1/5 for oxacillin and 4/17 for tobramycin) and mEs (16/19 for amikacin, 1/2 for tetracycline and 1/1 for clindamycin and penicillin G).

Seventy percent of the MICs reported were interpreted using EUCAST breakpoints and 30% were interpreted using CLSI breakpoints (Table 3). For all antimicrobials tested, the overall clinical categorical error rates that were due exclusively to the use of different breakpoints were 3.8% applying EUCAST breakpoints and 2.3% applying CLSI breakpoints (Table 3). The highest overall errors were observed with amikacin (25.0%), fosfomycin (14.3%), tobramycin (9.6%), oxacillin (6.6%) and gentamicin (5.7%), when EUCAST breakpoints where used, and quinupristin/dalfopristin (16.7%) and erythromycin (7.3%), when using CLSI breakpoints. The overall VMEs were 1.2% using EUCAST and 0.6% using CLSI. There were high rates of VMEs, particularly for linezolid (4.7% using EUCAST and 3.5% using CLSI), oxacillin (4.3% using EUCAST), tetracycline (1.8% using EUCAST and 3.0% using CLSI), clindamycin (1.6% using EUCAST and 3.6% using CLSI) and quinupristin/dalfopristin (5.6% using CLSI). The overall MEs were 1.8% with EUCAST and 0.7% with CLSI. The highest MEs using EUCAST were observed for fosfomycin (14.3%), tobramycin (9.1%) and gentamicin (5.7%) and when using CLSI were observed for quinupristin/dalfopristin (11.1%). The overall mEs were 0.8% using EUCAST and 1.0% using CLSI. The highest mEs occurred with amikacin (22.6% using EUCAST), followed by erythromycin (7.3% using CLSI).

### Type of reference isolate

As shown in Table 5, the highest percentages of discrepancies in MIC values were observed for isolates CC-07/*cfr* (48.0% for linezolid), CC-02/*mecC* (33.3% for oxacillin), CC-03/BORSA (25.5% for oxacillin) and CC-01/*mecA* (10.5% for cefoxitin and 6.1% for oxacillin). The discrepancies corresponded to VMEs or MEs (no mEs were observed). The highest percentages of VMEs were observed for CC-07/*cfr* with linezolid (33.3% and 28.6% using EUCAST and CLSI, respectively) and CC-04/MLSBi with clindamycin (12.5% and 33.3% using EUCAST and CLSI, respectively). In contrast, MEs were only observed for CC-03/BORSA with oxacillin (9.4% and 26.7% using EUCAST and CLSI, respectively).

**Table 5.** Distribution of discrepancies in MIC values and categorical error rates for *Staphylococcus* spp. by isolate

Isolate	Reference antimicrobials^a^	Discrepant MICs (%)^b^	No. of MICs interpreted using		Categorical errors (%)^c^			
					MEs		VMEs	
			EUCAST	CLSI	EUCAST	CLSI	EUCAST	CLSI
CC-01 (*mecA*)	Oxacillin	6.1	34	15	0.0	0.0	8.8	0.0
	Cefoxitin	**10.5**	16	3	0.0	0.0	12.5	0.0
CC-02 (*mecC*)	Oxacillin	**33.3**	33	15	0.0	0.0	18.2	0.0
	Cefoxitin	0.0	19	7	0.0	0.0	0.0	0.0
CC-03 (BORSA)	Oxacillin	**25.5**	32	15	**9.4**	**26.7**	0.0	0.0
	Cefoxitin	5.0	16	4	0.0	0.0	0.0	0.0
CC-04 (MLSBi)	Clindamycin	4.5	32	12	0.0	0.0	12.5	**33.3**
	Erythromycin	2.1	35	13	0.0	0.0	2.9	0.0
CC-05 (23S rRNA mutation)	Linezolid	0.0	33	14	0.0	0.0	0.0	0.0
CC-06 (*blaZ*)	Penicillin G	0.0	13	24	0.0	0.0	0.0	0.0
CC-07 (*cfr*)^d^	Linezolid	**48.0**	36	14	0.0	0.0	**33.3**	28.6
CC-08 (daptomycin non-susceptible)	Daptomycin	7.0	30	13	0.0	0.0	10.0	0.0

a The antimicrobial(s) used as phenotypic markers of resistance are analysed.

b Percentages of discrepancies in the MICs. MICs >1 dilution from the reference values were considered discrepant. MIC discrepancies >10% are highlighted in bold.

c mEs were not obtained. Overall errors were represented only by MEs or VMEs. The highest percentages of MEs and VMEs are highlighted in bold.

d Linezolid resistance.

### Ability of centres to infer resistance mechanisms

Thirty-nine out of 51 centres reported *S. aureus* CC-01/*mecA* as MRSA, *mecA* positive and/or modified PBP2a. Only 10 centres reported inference of *mecC* in *S. aureus* CC-02/*mecC*. With respect to the isolate CC-03/BORSA, only one centre indicated that this isolate had a BORSA phenotype. Nineteen centres reported macrolide-resistant *S. aureus* CC-04/MLSBi produced by a 23S rRNA methylase leading to the MLSBi resistance phenotype. Sixteen of these 19 centres further indicated MLSBi resistance and 3 centres indicated constitutive MLSB (MLSB_c_) resistance. Only three centres associated linezolid resistance with the *S. epidermidis* CC-05/23S rRNA mutation isolate with the presence of mutations in 23S rRNA. Penicillinase production by *S. aureus* CC-06/*blaZ* was reported by 21 centres. In the linezolid-resistant *S. aureus* CC-07/*cfr* isolate, the presence of the *cfr* gene was reported by only eight centres. Daptomycin non-susceptibility in isolate CC-08/daptomycin non-susceptible was reported by 19 centres.

## Discussion

The results obtained in the present study show that the discrepancies in MIC values and error rates in categorization observed for some antimicrobials were related to the AST system used, the application of EUCAST or CLSI breakpoints, the antimicrobial agent and the type of isolate.

Accurate AST of *Staphylococcus* spp. is essential for guiding therapy as well as for surveillance of antimicrobial resistance and for rapid implementation of appropriate infection control measures. The use of automated AST systems can produce false-susceptible and false-resistant results, which can lead to serious implications in the clinical outcome of infected patients. In this study, gradient diffusion and in-house broth microdilution were the least reliable AST systems, due to the unacceptably high percentage of VMEs using gradient strips and MEs using in-house broth microdilution. Not every laboratory investigated the reason for these findings, although it may have been related to the inoculum preparation, the method used to determine MIC endpoints, or the management of colonies within the inhibition zone.^22^

Gradient strips produced 4.3% to 5.1% of false-susceptible results (VMEs), which occurred mainly with linezolid. This can represent a serious problem because susceptible linezolid MIC results are not routinely confirmed in the microbiology laboratory. Failing to identify linezolid-resistant MRSA isolates may lead to treatment failures that could be associated with increased mortality, prolonged length of stay, reinfections and undetected outbreaks. The non-recognition of linezolid-resistant *cfr*-positive MRSA may have important epidemiological repercussions, as this resistance determinant is plasmid-encoded and can be transferred to linezolid-susceptible *Staphylococcus* spp. and *Enterococcus* spp.

In-house broth microdilution, in contrast to gradient strips, produced 4.8% of MEs, which were associated with only one result of false resistance to oxacillin. This result should be interpreted with caution due to the small number of determinations reported using this AST method. False resistance to oxacillin in *S. aureus* is more likely to be confirmed in the microbiology laboratory using additional phenotypic or molecular tests that can detect the presence of the modified PBP2a (e.g. by latex agglutination or immunochromatography) or the *mecA* gene (e.g. by PCR).

Regarding the automated systems used for AST in this study, the least reliable one was MicroScan, due to the high percentage of VMEs (particularly for oxacillin and linezolid), MEs (particularly for gentamicin and tobramycin) and mEs (particularly for amikacin). The most relevant errors obtained using the other two automated AST systems occurred with VITEK 2 and erythromycin (VMEs, ME and mEs) and with Phoenix and linezolid (VME). It is important to highlight that unexpected MIC results or MICs associated with clinically relevant resistance for a particular antimicrobial, such as oxacillin for MRSA, linezolid, glycopeptides or daptomycin, obtained with some of these AST systems should be retested or confirmed with a secondary MIC-based method, due to the potential clinical and epidemiological impact of these resistances.

EUCAST breakpoints were more frequently used by the participating laboratories than CLSI breakpoints, reflecting the current trend in Europe towards the implementation of EUCAST guidelines. Migration from CLSI to EUCAST recommendations has important microbiological, clinical and epidemiological advantages and disadvantages, as there are relevant differences between the two committees that can affect the interpretation of the MIC results obtained.^17^^,^^22^^,^^23^

With respect to the breakpoints, the percentage of overall errors was higher using EUCAST than using CLSI. This is probably related to the I category, which is not defined for various antimicrobials by EUCAST (e.g. glycopeptides, linezolid or daptomycin) and/or CLSI (e.g. fosfomycin), the wide MIC interval of the I category for some antimicrobials using CLSI (e.g. glycopeptides, aminoglycosides, clindamycin and erythromycin) and the lower breakpoints defined by EUCAST for various antimicrobials (e.g. ciprofloxacin, levofloxacin, vancomycin, teicoplanin, gentamicin, tobramycin and tetracycline) with respect to CLSI (see below).

The percentages of MEs and VMEs using EUCAST were slightly higher than those obtained using CLSI, whereas there was a similar, relatively low percentage of mEs (<10%), which was considered acceptable and probably without significant microbiological and clinical relevance.^22^ The percentages of VMEs and MEs observed for some antimicrobials (e.g. clindamycin, gentamicin, tobramycin and amikacin) may be attributable to some factors, such as the former I clinical category, which is defined by CLSI for some of these antimicrobials but not by EUCAST, the absence of defined breakpoints (e.g. fosfomycin, amikacin and tobramycin using CLSI) and the breakpoints used, which are ≥1 dilution lower by EUCAST than by CLSI.^17^^,^^22^^,^^23^ In contrast, for other much more clinically relevant antimicrobials, such as oxacillin and linezolid, the differences observed could not be attributable to the former I clinical category, because for these agents EUCAST and CLSI do not have this clinical category and the breakpoints defined by EUCAST are the same as those defined by CLSI. Although the causes of the MIC discrepancies and categorical errors observed with oxacillin and linezolid remain unknown, these differences cannot be dismissed as being related to the bacterial inoculum preparation or the interpretation of the MICs.^22^

The type of antimicrobial was another factor contributing to MIC discrepancies and categorical errors. The most clinically relevant antimicrobials that showed the greatest MIC discrepancies and/or categorical error rates were daptomycin, oxacillin and linezolid.

Daptomycin was the antimicrobial with the highest discrepant MIC values, which were mainly obtained by VITEK 2 and MicroScan and were associated with a low percentage of VMEs (1.3%) and MEs (0.4%). These discrepancies could be related to the inoculum preparation or the calcium content in the medium, as previously described.^24^ The very low percentage of MEs should not have any clinical relevance whereas the VMEs may be of great relevance, especially in severe infections such as MRSA endocarditis, where the treatment options are limited and therapeutic failures may occur.^24^

In our study, we detected an unacceptably high percentage of discrepant MICs of oxacillin, which were predominantly obtained with MicroScan. Nearly 20% of these discrepancies accounted for most of the VMEs obtained (4.3% using EUCAST breakpoints), which contrasts with previous studies reporting up to 6.7% of VMEs using MicroScan and 14.2% of VMEs using VITEK 2.^25^ Most of these MIC discrepancies observed with oxacillin occurred especially with CC-02/*mecC* and, to a lesser extent, CC-01/*mecA* isolates. As mentioned before, false-susceptible results are very difficult to detect in the laboratory because susceptible results are not usually confirmed by an alternative AST method. The lack of recognition of false oxacillin-susceptible results can have very important repercussions from a clinical and an epidemiological point of view. First, the use of oxacillin in infections caused by MRSA isolates, particularly if they are biofilm-forming, can be associated with a high probability of therapeutic failure with fatal consequences, absence of implementation of contact precautions and a bad clinical outcome for infected patients.^26^ Second, true MRSA isolates reported as susceptible to oxacillin may contribute to the emergence of nosocomial outbreaks caused by MRSA.

Around 5% of the discrepancies in the MICs of oxacillin produced most of the MEs observed, which were relatively low (1.9%–3.4%). Oxacillin resistance can be difficult to detect, especially when the *mecA* gene is heterogeneously expressed or when oxacillin resistance is due to hyperproduction of β-lactamases or point mutations in PBP genes (BORSA isolates).^5^^,^^6^^,^^27^ Furthermore, *mecA* gene expression is affected by many factors, including temperature, incubation time, medium used and sodium chloride concentration, which can contribute to unreliable oxacillin MICs.^2^^,^^3^^,^^28^ The main clinical repercussion of reporting false oxacillin resistance in staphylococci lies in the use of other antimicrobials, such as glycopeptides, with a broader spectrum than oxacillin and probably with more toxic effects. Furthermore, with respect to the epidemiological consequences, particularly with *S. aureus*, this leads to the adoption of unnecessary contact precautions.

Most discrepancies in the MICs of linezolid were obtained using MicroScan and, to a lesser extent, gradient strips. These discrepancies were acceptable but about half of them were responsible for an unacceptable 4.7% of VMEs obtained by EUCAST. Most of these VMEs occurred with the CC-07/*cfr* isolate and they were probably related to the loss of the plasmid encoding *cfr*, but this possibility was not investigated. False linezolid susceptibility results, as happened with oxacillin, are difficult to recognize in the laboratory due to the fact that MIC results associated with susceptibility are infrequently confirmed. In addition, these false results of linezolid susceptibility can have an important clinical impact, because of the possibility of therapeutic failures, and epidemiological relevance, due to the potential ability of the plasmid-encoded *cfr* to spread and to be transferred to linezolid-susceptible isolates of *Staphylococcus* spp. and *Enterococcus* spp.

Other antimicrobials for which unacceptable MIC discrepancies and categorical errors were obtained, but with less clinical relevance than oxacillin, daptomycin or linezolid, included quinupristin/dalfopristin, clindamycin, fosfomycin and the aminoglycosides gentamicin, tobramycin and amikacin.

Discrepancies in the MICs of gentamicin, tobramycin and amikacin were very similar and relatively low (7.0%–8.1%). The MEs for gentamicin (5.7%) and tobramycin (9.1%) and the mEs for amikacin (22.6%) were mostly obtained using MicroScan and were unacceptable. For gentamicin, the MEs observed were only produced by discrepant MICs, whereas for amikacin and tobramycin there was a significant contribution from non-discrepant MICs in the errors obtained (MEs for tobramycin and mEs for amikacin). Probably, most of these MEs and mEs may be explained, at least in part, by the presence of residual or minimal growth in the bottom of wells containing aminoglycoside concentrations higher than the MIC, as previously reported for tobramycin and *Acinetobacter baumannii*.^29^ If this scant growth were taken into account (false positive or ME), it would be expected to result in higher MIC values and so increase the number of discrepant results. This hypothesis is in agreement with the false-resistance results reported for gentamicin and tobramycin, whereas for amikacin it is supported by the high mEs of the RI (resistant/intermediate) type (R by the participating centre, I by the reference laboratories) and IS (intermediate/susceptible) type (I by the participating centre and S by the reference laboratories). The elevated percentage of mEs and MEs obtained for the aminoglycosides tested were observed using MicroScan. The clinical impact of the MICs associated with these errors could be minimized if they were confirmed in the laboratory by other AST systems, like disc diffusion or gradient strips. It is also important to note that these categorical errors with aminoglycosides should not have a significant clinical impact since these antimicrobials are not frequently used as first-line treatment in infections caused by *Staphylococcus* spp.

The discrepancies in the MICs of clindamycin and tetracycline were quite different (13.5% for clindamycin and 3.5% for tetracycline) and they were associated with the use of MicroScan. The errors produced by these discrepant MICs were very similar (1.6%–3.6% of VMEs for clindamycin and 1.9%–3.0% of VMEs for tetracycline). For clindamycin, these discrepancies could be explained by those centres that did not perform the D-test assay for the detection of inducible clindamycin resistance, which is in agreement with the finding that all the VMEs observed for clindamycin were caused by non-discrepant MICs. The clinical impact of these VMEs obtained for clindamycin and tetracycline should have little clinical relevance, as these antimicrobials are not frequently used as first-line therapy for the treatment of infections caused by *Staphylococcus* spp.

The very high MIC discrepancies observed for fosfomycin were associated with the use of VITEK 2 and they caused an unacceptable, very high percentage of MEs (18.6%). Nevertheless, these results should be interpreted with caution due to the low number of fosfomycin MIC determinations reported in this study. These MIC discrepancies may have been due to the inoculum preparation and selection of resistant subpopulations, as previously described.^15^ The majority of automated AST systems should not be used for testing fosfomycin susceptibility, as they provide unreliable results compared with the reference agar dilution gold standard method.^30^^,^^31^ False resistance to fosfomycin can be detected in the laboratory by the agar dilution method, but this method is difficult to perform and requires the addition of glucose-6-phosphate to the medium. As an alternative to the agar dilution method, there is a rapid commercial agar dilution panel available, recently evaluated by Campanile *et al*.^32^ The clinical impact of the VMEs observed with fosfomycin may be relatively low since this antimicrobial is not frequently used as first-line therapy for the treatment of infections caused by *Staphylococcus* spp. due to the high selection rates of fosfomycin-resistant mutants, particularly if it is used as monotherapy.

With respect to quinupristin/dalfopristin, the discrepancies in MIC values were acceptable, although the rates of MEs and VMEs were unacceptably high. These errors were only observed when applying CLSI breakpoints, but not with EUCAST, indicating that VMEs and MEs for this combination of antimicrobials should not represent a serious problem from a therapeutic point of view. Furthermore, in Europe at least, quinupristin/dalfopristin is infrequently used in clinical practice for the treatment of infections caused by MRSA.^33^

With respect to the type of isolate, the most important discrepancies and errors occurred in the linezolid-resistant isolate CC-07/*cfr* and among the MRSA isolates, CC-01/*mecA* and CC-02/*mecC.* The discrepancies in the MICs of linezolid for CC-07/*cfr* were extremely high (33.3%), were associated with the use of MicroScan, and produced an unacceptably high rate of VMEs (33.3%). As previously stated, these discrepancies could probably be explained by the loss of the plasmid encoding the *cfr* resistance determinant, although it has also been stated that *cfr*-positive isolates can show very low MICs, below the resistance breakpoint, and are hardly detected when using gradient diffusion tests.^34^

The MIC discrepancies observed for the CC-01/*mecA* isolate with cefoxitin and oxacillin were associated with the use of MicroScan, accounting for two VMEs for cefoxitin and three VMEs for oxacillin. Regarding isolate CC-02/*mecC*, the discrepancies obtained could have been associated with the categorical errors obtained for oxacillin obtained by MicroScan (5 out of 6 VMEs reported), indicating the low sensitivity of oxacillin for the detection of MRSA isolates carrying the *mecC* gene, as reported in previous studies.^35^

The number of laboratories able to infer some type of resistance mechanism(s) was low, particularly for certain antimicrobials such as oxacillin. Inference of this kind is complicated by the nature of the resistance mechanism (e.g. heterogeneous expression, low level of expression, inducible resistance etc.). In our study, a major discrepancy was found when inferring resistance mechanisms, particularly with isolates CC-07/*cfr*, CC-02/*mecC* and CC-03/BORSA.

An analysis of potential factors that contribute to erroneous antimicrobial susceptibility results should be a priority for clinical laboratories. One way to address this problem is to participate in quality control programmes, which can be very helpful in detecting potential laboratory problems and enabling corrective measures to be established for optimizing the process and the quality of the reports offered to clinicians. This information is very useful for optimizing the best therapeutic strategies, improving the rational use of antimicrobials (reducing resistance rates), facilitating the control of nosocomial infections (by reducing spread of MDR clones) and preventing outbreaks.^36–41^

The main limitations of the present study are its small sample size, which is typical of this kind of study, and the probable overestimation of MIC discrepancies and categorical errors due to the special characteristics of the isolates selected for this study.

In conclusion, this study shows that microbiology laboratories in Spain need to improve their ability to accurately determine the antimicrobial susceptibility of *Staphylococcus* spp. This is particularly important for antimicrobials frequently used in clinical settings, such as oxacillin, linezolid and aminoglycosides, and when both automated systems and EUCAST breakpoints are used.

## References

[1] ECDC. Healthcare-associated Infections Acquired in Intensive Care Units - Annual Epidemiological Report for 2017. 2019. https://www.ecdc.europa.eu/en/publications-data/healthcare-associated-infections-intensive-care-units-annual-epidemiological-1.

[2] Peacock SJ , Paterson GK. Mechanisms of methicillin resistance in *Staphylococcus aureus*. Annu Rev Biochem 2015; 84: 577. 26034890 10.1146/annurev-biochem-060614-034516

[3] Chambers HF. Methicillin resistance in staphylococci: molecular and biochemical basis and clinical implications. Clin Microbiol Rev 1997; 10: 781–91. 9336672 10.1128/cmr.10.4.781 PMC172944

[4] García-Álvarez L , Holden MT, Lindsay H et al Meticillin-resistant *Staphylococcus aureus* with a novel *mecA* homologue in human and bovine populations in the UK and Denmark: a descriptive study. Lancet Infect Dis 2011; 11: 595–603. 21641281 10.1016/S1473-3099(11)70126-8 PMC3829197

[5] Skinner S , Murray M, Walus T et al Failure of cloxacillin in treatment of a patient with borderline oxacillin-resistant *Staphylococcus aureus* endocarditis. J Clin Microbiol 2009; 47: 859–61. 19116360 10.1128/JCM.00571-08 PMC2650896

[6] Hryniewicz MM , Garbacz K. Borderline oxacillin-resistant *Staphylococcus aureus* (BORSA)—a more common problem than expected? J Med Microbiol 2017; 66: 1367–73. 28893360 10.1099/jmm.0.000585

[7] ECDC. Surveillance of Antimicrobial Resistance in Europe 2018. 2019. https://www.ecdc.europa.eu/en/publications-data/surveillance-antimicrobial-resistance-europe-2018.

[8] Leclercq R. Mechanisms of resistance to macrolides and lincosamides: nature of the resistance elements and their clinical implications. Clin Infect Dis 2002; 34: 482–92. 11797175 10.1086/324626

[9] Tsiodras S , Gold HS, Sakoulas G et al Linezolid resistance in a clinical isolate of *Staphylococcus aureus*. Lancet 2001; 358: 207–8. 11476839 10.1016/S0140-6736(01)05410-1

[10] Morales G , Picazo JJ, Baos E et al Resistance to linezolid is mediated by the *cfr* gene in the first report of an outbreak of linezolid-resistant *Staphylococcus aureus*. Clin Infect Dis 2010; 50: 821–5. 20144045 10.1086/650574

[11] Long KS , Poehlsgaard J, Kehrenberg C et al The cfr rRNA methyltransferase confers resistance to phenicols, lincosamides, oxazolidinones, pleuromutilins, and streptogramin A antibiotics. Antimicrob Agents Chemother 2006; 50: 2500–5. 16801432 10.1128/AAC.00131-06 PMC1489768

[12] Barros EM , Martin MJ, Selleck EM et al Daptomycin resistance and tolerance due to loss of function in *Staphylococcus aureus dsp1* and *asp23*. Antimicrob Agents Chemother 2018; 63: e01542-18. 30397055 10.1128/AAC.01542-18 PMC6325204

[13] Swenson JM , Spargo J, Tenover FC et al Optimal inoculation methods and quality control for the NCCLS oxacillin agar screen test for detection of oxacillin resistance in *Staphylococcus aureus*. J Clin Microbiol 2001; 39: 3781–4. 11574618 10.1128/JCM.39.10.3781-3784.2001 PMC88434

[14] Nonhoff C , Rottiers S, Struelens MJ. Evaluation of the Vitek 2 system for identification and antimicrobial susceptibility testing of *Staphylococcus* spp. Clin Microbiol Infect 2005; 11: 150–3. 15679491 10.1111/j.1469-0691.2004.01047.x

[15] Ballestero-Téllez M , Docobo-Pérez F, Rodríguez-Martínez JM et al Role of inoculum and mutant frequency on fosfomycin MIC discrepancies by agar dilution and broth microdilution methods in Enterobacteriaceae. Clin Microbiol Infect 2017; 23: 325–31. 28062317 10.1016/j.cmi.2016.12.022

[16] Heil EL , Johnson JK. Impact of CLSI breakpoint changes on microbiology laboratories and antimicrobial stewardship programs. J Clin Microbiol 2016; 54: 840–4. 26791363 10.1128/JCM.02424-15 PMC4809933

[17] Larrosa MN , Benito N, Cantón R et al From CLSI to EUCAST, a necessary step in Spanish laboratories. Enferm Infecc Microbiol Clin 2020; 38: 79–83. 10.1016/j.eimc.2018.09.014 30409509

[18] Livermore DM , Winstanley TG, Shannon KP. Interpretative reading: recognizing the unusual and inferring resistance mechanisms from resistance phenotypes. J Antimicrob Chemother 2001; 48: 87–102. 11420342 10.1093/jac/48.suppl_1.87

[19] Cantón R. Interpretive reading of the antibiogram: a clinical necessity. Enferm Infecc Microbiol Clin 2010; 28: 375–85. 20381926 10.1016/j.eimc.2010.01.001

[20] CLSI. *Performance Standards for Antimicrobial Susceptibility Testing—Twenty-Eighth Edition: M100*. 2018.

[21] EUCAST. Clinical Breakpoints and Epidemiological Cut-Off Values. Version 8.1. 2018. https://www.eucast.org/fileadmin/src/media/PDFs/EUCAST_files/Breakpoint_tables/v_8.1_Breakpoint_Tables.pdf.

[22] Humphries RM , Ambler J, Mitchell SL et al CLSI Methods Development and Standardization Working Group best practices for evaluation of antimicrobial susceptibility tests. J Clin Microbiol 2018; 56: e01934-17. 29367292 10.1128/JCM.01934-17 PMC5869819

[23] Cusack TP , Ashley EA, Ling CL et al Impact of CLSI and EUCAST breakpoint discrepancies on reporting of antimicrobial susceptibility and AMR surveillance. Clin Microbiol Infect 2019; 25: 910–1. 30910717 10.1016/j.cmi.2019.03.007 PMC6587648

[24] Humphries RM , Pollett S, Sakoulas G. A current perspective on daptomycin for the clinical microbiologist. Clin Microbiol Rev 2013; 26: 759–80. 24092854 10.1128/CMR.00030-13 PMC3811228

[25] Skulnick M , Simor AE, Gregson D et al Evaluation of commercial and standard methodology for determination of oxacillin susceptibility in *Staphylococcus aureus*. J Clin Microbiol 1992; 30: 1985–8. 1500504 10.1128/jcm.30.8.1985-1988.1992 PMC265428

[26] Deurenberg RH , Vink C, Kalenic S et al The molecular evolution of methicillin-resistant *Staphylococcus aureus*. Clin Microbiol Infect 2007; 13: 222–35. 17391376 10.1111/j.1469-0691.2006.01573.x

[27] de Lencastre H , Sá Figueiredo AM, Urban C et al Multiple mechanisms of methicillin resistance and improved methods for detection in clinical isolates of *Staphylococcus aureus*. Antimicrob Agents Chemother 1991; 35: 632–9. 2069369 10.1128/aac.35.4.632 PMC245071

[28] Berger-Bächi B , Rohrer S. Factors influencing methicillin resistance in staphylococci. Arch Microbiol 2002; 178: 165–71. 12189417 10.1007/s00203-002-0436-0

[29] Moodley VM , Oliver SP, Shankland I et al Evaluation of five susceptibility test methods for detection of tobramycin resistance in a cluster of epidemiologically related *Acinetobacter baumannii* isolates. J Clin Microbiol 2013; 51: 2535–40. 23698528 10.1128/JCM.03250-12 PMC3719664

[30] EUCAST. Routine and extended internal quality control for MIC determination and disk diffusion as recommended by EUCAST. Version 10.0, valid from 01/01/2020. 2020. https://www.eucast.org/fileadmin/src/media/PDFs/EUCAST_files/QC/v_10.0_EUCAST_QC_tables_routine_and_extended_QC.pdf

[31] CLSI. *Methods for Dilution Antimicrobial Susceptibility Tests for Bacteria that Grow Aerobically—Eleventh Edition: M07*. 2018.

[32] Campanile F , Wootton M, Davies L et al Gold standard susceptibility testing of fosfomycin in *Staphylococcus aureus* and Enterobacterales using a new agar dilution panel^®^. J Glob Antimicrob Resist 2020; 23: 334–7. 33127523 10.1016/j.jgar.2020.08.025

[33] Drew RH , Perfect JR, Srinath L et al Treatment of methicillin-resistant *Staphylococcus aureus* infections with quinupristin–dalfopristin in patients intolerant of or failing prior therapy. J Antimicrob Chemother 2000; 46: 775–84. 11062197 10.1093/jac/46.5.775

[34] Arias CA , Vallejo M, Reyes J et al Clinical and microbiological aspects of linezolid resistance mediated by the *cfr* gene encoding a 23S rRNA methyltransferase. J Clin Microbiol 2008; 46: 892–6. 18174304 10.1128/JCM.01886-07 PMC2268374

[35] Kriegeskorte A , Idelevich EA, Schlattmann A et al Comparison of different phenotypic approaches to screen and detect *mecC*-harboring methicillin-resistant *Staphylococcus aureus*. J Clin Microbiol 2017; 56: e00826-17. 28978682 10.1128/JCM.00826-17 PMC5744205

[36] Fernández-Cuenca F , Tomás M, Caballero-Moyano FJ et al Reporting antimicrobial susceptibilities and resistance phenotypes in *Acinetobacter* spp: a nationwide proficiency study. J Antimicrob Chemother 2018; 73: 692–7. 29244131 10.1093/jac/dkx464

[37] Bronzwaer S , Buchholz U, Courvalin P et al Comparability of antimicrobial susceptibility test results from 22 European countries and Israel: an external quality assurance exercise of the European Antimicrobial Resistance Surveillance System (EARSS) in collaboration with the United Kingdom National External Quality Assurance Scheme (UK NEQAS). J Antimicrob Chemother 2002; 50: 953–64. 12461017 10.1093/jac/dkf231

[38] Cantón R , Loza E, del Carmen Conejo M et al Quality control for β-lactam susceptibility testing with a well-defined collection of Enterobacteriaceae and *Pseudomonas aeruginosa* strains in Spain. J Clin Microbiol 2003; 41: 1912–8. 12734226 10.1128/JCM.41.5.1912-1918.2003 PMC154698

[39] Chaitram JM , Jevitt LA, Lary S et al The World Health Organization’s External Quality Assurance System Proficiency Testing Program has improved the accuracy of antimicrobial susceptibility testing and reporting among participating laboratories using NCCLS methods. J Clin Microbiol 2003; 41: 2372–7. 12791851 10.1128/JCM.41.6.2372-2377.2003 PMC156569

[40] Jones RN , Glick T, Sader HS et al Educational antimicrobial susceptibility testing as a critical component of microbiology laboratory proficiency programs: American Proficiency Institute results for 2007-2011. Diagn Microbiol Infect Dis 2013; 75: 357–60. 23481025 10.1016/j.diagmicrobio.2013.01.027

[41] Tenover FC , Mohammed MJ, Stelling J et al Ability of laboratories to detect emerging antimicrobial resistance: proficiency testing and quality control results from the World Health Organization’s external quality assurance system for antimicrobial susceptibility testing. J Clin Microbiol 2001; 39: 241–50. 11136778 10.1128/JCM.39.1.241-250.2001 PMC87709

